# Antireflux endoscopic injection therapy in post-pubertal patients via techniques adopted for the dilated ureteral orifice: a retrospective single-center study

**DOI:** 10.1186/s12894-021-00842-3

**Published:** 2021-04-24

**Authors:** Tetsuji Maruyama, Kentaro Mizuno, Hidenori Nishio, Taiki Kato, Takashi Hamakawa, Yosuke Ikegami, Takahiro Yasui, Yutaro Hayashi

**Affiliations:** 1 Department of Urology, Nagoya City East Medical Center, 2 Wakamizu-cho, Chikusa-ku, Nagoya, 464-8547 Japan; 2 Department of Pediatric Urology, Nagoya City University Graduate School of Medical Sciences, 1 Kawasumi, Mizuho-cho, Mizuho-ku, Nagoya, 467-8601 Japan; 3 Department of Nephro-Urology, Nagoya City University Graduate School of Medical Sciences, 1 Kawasumi, Mizuho-cho, Mizuho-ku, Nagoya, 467-8601 Japan

**Keywords:** Dextranomer/hyaluronic acid copolymer, HIT, STING, Urinary tract infection, Vesicoureteral reflux

## Abstract

**Background:**

To investigate the efficacy and safety of endoscopic injection therapy for vesicoureteral reflux in post-pubertal patients with dilated ureteral orifice via modified hydrodistension implantation techniques.

**Methods:**

We retrospectively reviewed medical records including operational procedure and clinical course of all consecutive patients over 12 years old with a history of injection therapy. Endoscopic injection of dextranomer/hyaluronic acid copolymer was performed under hydrodistension implantation technique with some modifications in order to inject through dilated ureteral orifice align with the intramural ureter. Technical selections were done according to hydrodistension grade of the ureteral orifice. Voiding cystourethrography was evaluated at 3 months postoperatively. Hydronephrosis was evaluated using ultrasonography preoperatively until 6 months postoperatively.

**Results:**

From 2016 to 2019, 12 patients (all female, 16 ureteral units; median age 32 [range 15–61] years) underwent endoscopic injection therapy at one of our institutions. We have identified grade II vesicoureteral reflux in 5 ureters, grade III in 8, and grade IV in 3 ureters. Grade 3 ureteral-orifice dilation were presented in 12 ureters (75%), grade 2 in 3 and grade 1 in 1 ureter in the present cases. Postoperatively, vesicoureteral reflux was diminished to grade 0 in 12 ureteral units (75%), decreased to grade I in 3 (9%), and remained grade III in 1 (6%). Three patients reported dull flank pain for several days postoperatively and there was 1 case of acute pyelonephritis. Temporary hydronephrosis was confirmed in 3 ureteral units (19%) at 1 month postoperatively. Median follow-up duration was 23 (range 13–63) months long. Although, 3 patients were experienced f-UTI 1–2 times, repeated VCUG showed no VUR recurrence.

**Conclusions:**

According to hydrodistension grade of the ureteral orifice, endoscopic injection therapy via modified hydrodistension implantation technique is an effective and safe treatment for vesicoureteral reflux in post-pubertal female patients with dilated ureteral orifice. While ureteral deformities or a history of anti-reflux surgery may increase the risks, these can be managed with appropriate methods that ensure sufficient mound appearance and height.

## Background

Vesicoureteral reflux (VUR) is major cause of febrile urinary tract infection (f-UTI) in children and sometimes adolescent and young adult females [1, 2]. For ureteral reimplantation, open surgery is the gold standard procedure owing to its reliably high success rate (more than 95%) [3, 4]. However, the development of a less invasive approach such as laparoscopic or robot-assisted surgery would be highly arising in the field [5]. In addition, the use of endoscopic injection therapy has increased since the injectable agents and techniques have been refined, resulting in subureteral transurethral injection (STING), hydrodistension implantation technique (HIT), and double-HIT procedure [6–8]. Currently, this minimally invasive therapy is a new option for the treatment of VUR in children [9].

In adult patients, less invasiveness is also eligible so that impact of the surgery to their active social life are sever. However, there is little information regarding the clinical utility of the therapy partially due to the limited number of patients or such technical difficulties associated with large-caliber ureter or might be pathological stiffness originated from chronic inflammation in the adult ureter.

The present study investigated the efficacy and safety of endoscopic injection therapy for VUR in adults via modified HIT techniques adopted for large-caliber ureters. The primary objective was the successful treatment of VUR defined as the absence of VUR at 3-month follow-up by voiding cystourethrography (VCUG). The secondary objectives were complications including ureteral obstruction or occurrence of comorbidity such as flank pain or renal failure caused by hydronephrosis or f-UTI. And also estimated were factors that might contribute to the successful treatment so that grade and timing of VUR and hydrodistention (HD) grade [7].

## Methods

### Preoperative evaluation

After institutional review board approval (20-04-330), we retrospectively reviewed medical records of all consecutive patients over 12 years old who were referred to one of our facilities (Nagoya City East Medical Center) between 2016 and 2019 underwent endoscopic injection therapy for VUR. All patients were referred from our tertiary center (Nagoya City University) and were willing to undergo injection therapy after discussion of all surgical options (open, laparoscopic, and robot-assisted-surgery) along with their advantages and disadvantages [10, 11]. After explaining the possible outcomes, complications, and comorbidities associated with each procedure, written informed consent was obtained from all patients before the surgery. In case of subjects are under 18, the written informed consent was also obtained from a parent and/or legal guardian.

All patients presented with several episodes of f-UTI were include in the study. Patients with a history of any anti-reflux surgery were not excluded. Exclusion criteria were grade V VUR, grade I VUR without contralateral VUR, ureterocele, posterior urethral valves, obstructed megaureter and ectopic ureters, and presence of voiding dysfunction. The reflux grade was based on the results of preoperative VCUG, according to the International Classification System (International Reflux Study Committee), which was also evaluated postoperatively. We re-evaluated VCUG before surgery to confirm grade and timing (i.e., filling or voiding). Hydronephrosis was evaluated using ultrasonography preoperatively and until 6 months postoperatively. The hydronephrosis was graded according to the Society for Fetal Urology. None of the patients in this series exhibited voiding dysfunction at the time of injection, as confirmed by uroflowmetry just before and after surgery.

### Surgical procedure

All procedures were performed at one facility by a single surgeon (TM) using one material; dextranomer/hyaluronic acid copolymer (Dx/HA). The surgeon had previously conducted endoscopic injection surgery using another material (Teflon) with two of the other authors (TY, YH) [12].

Cystography was conducted first under general or lumbar anesthesia. If reflux was observed, cystography was repeated intraoperatively. A pediatric urethral cystoscope with an offset lens (8-12Fr, 13 cm long; KARL STORZ K27030KA) was placed in position and the configuration of the ureteral orifice recorded. Subsequently, HD was performed to grade the dilation of the ureteral orifice under irrigation 40 cm above the patient’s position but not exceeded 50% of the expected bladder capacity [7]. HD technique was originally introduced by Kirsh et al. [7], which direct a pressure stream of irrigation fluid into the ureter (hydrodistension) to define the site of injection within ureteral submucosa. HD grade was recorded according to the definition [13]. Briefly, H3 is defined as ‘orifice opens and extramural ureter evident’, H2; orifice opens and intramural tunnel evident, H1; orifice opens and intramural tunnel not evident, H0; no orifice distention evident. Although we recorded the contralateral HD grade, we did not perform prophylactic injection for single-sided VUR as is not approved by the Japanese healthcare insurance. Endoscopic injection was performed via STING [6], HIT [7], or double-HIT [8] methods, with some modifications adopted for large -caliber adult ureter (described below).

**Fig. 1:**
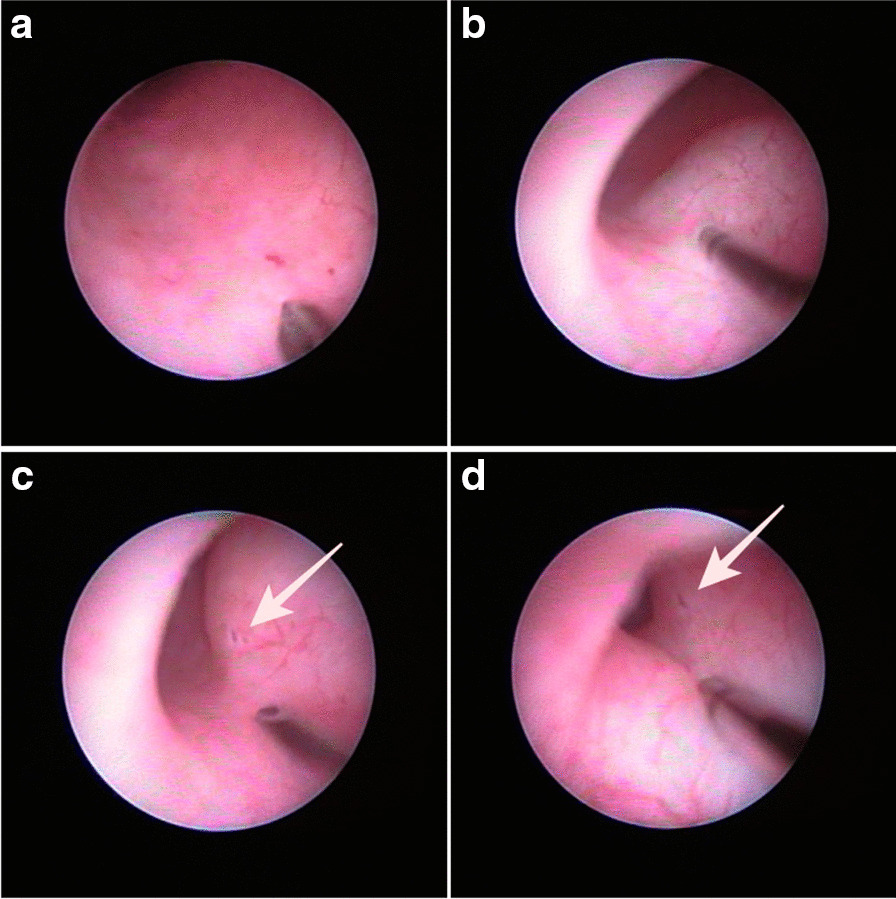
Intraoperative photographs of inserting hydrodistension implantation technique (I-HIT). (**a**) First injection inside intramural ureter, (**b**) after small amount injected, (**c**) second injection near the first (arrow), (**d**) after second injection

**Fig. 2:**
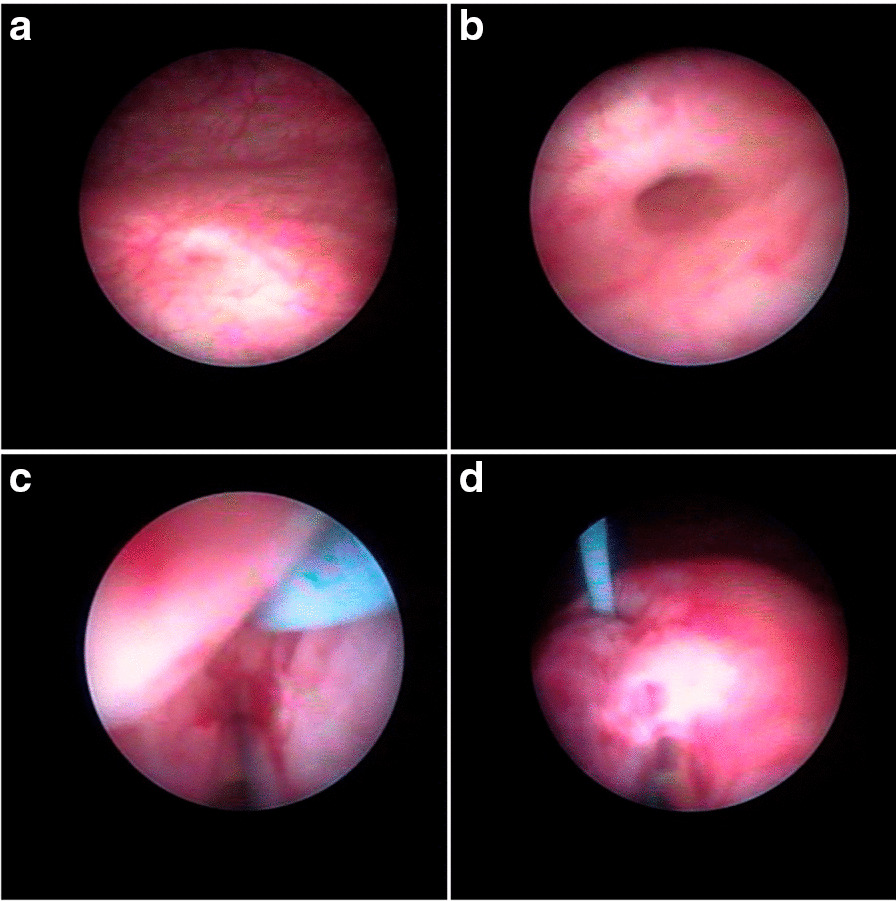
Intraoperative photograph of guide-wired hydrodistension implantation technique (G-HIT). (**a**) The ureteral orifice looked like horseshoe and (**b**) Hydrodistension grade 2 appearance under hydrodistension, (**c**) injection using guidewire, (**d**) combined subureteral injection technique after the injection

Injection method was selected according to HD grade. Briefly, in cases determined to be grade H3 when the cystoscope could be inserted through the intramural ureter, injection was performed confirming direction of injection needle align with the ureter and punctured at the proximal and distal portion without withdrawing the scope (referred to as inserting HIT/I-HIT) (Fig. 1) until a high mound formed. In cases graded as H2, a guide wire was used to inject align to ureteral direction at the distal portion (referred to as guide-wired HIT/G-HIT) (Fig. 2). To create a volcano-like mound [14], Dx/HA was added in cases where STING was performed. In all procedures, the total injection volume was kept below 3.0 ml per ureter.

### Postoperative evaluation

Success was defined as the absence of VUR as on VCUG at the 3-month follow-up. Following successful treatment of VUR by Dx/HA injection, antibiotic prophylaxis was discontinued, and patients were followed-up with regular urinalysis and ultrasound until 1 year postoperatively. A repeat VCUG was performed after successful endoscopic treatment when the patient had previously experienced at least one episode of f-UTI or repeated afebrile UTIs. Other adverse events including flunk pain, low abdominal pain, dysuria, or low- or high-grade fever were evaluated.

### Statistical analysis

The Chi-square test or Fisher’s exact test were used to compare two groups with respect to a dichotomous endpoint. The Mann–Whitney test was used to compare two groups with respect to a continuous endpoint such as injection time or volume. The Kruskal–Wallis H-test was used for comparisons between three groups. Statistical significance was set as *P*-value < 0.05. All reported p values are two-sided. All statistical analyses were performed using R statistical software (version 3.2.3, The R Foundation for Statistical Computing, Vienna, Austria).

## Results

### Preoperative evaluation

Twelve post-pubertal patients (all female, a total of 16 ureteral units) with a median age of 32 (range 15–61) years underwent endoscopic injection therapy at Nagoya East Medical Center (Table 1). Four patients had bilateral VUR, the other 8 exhibited single-sided VUR. The VUR was evaluated to be grade II in 5 ureters, grade III in 8 and grade IV in 3. Two patients had a history of anti-reflux surgery; one had undergone collagen injection remained single-sided grade III VUR, the other had undergone laparoscopic implantations by extravesical approach remained single -sided grade II VUR.

**Table 1:** Patients data

Age (y), median (range)	32 (15–61)
Gender, n (%)	
Female	12 (100)
Male	0 (0)
Affected side, n	
Right	8
Left	8
Laterality, n	
Single side	8
Bilateral	4
VUR grade, n [ureteral units]	
II	5
III	8
IV	3
Indications for operation, n (%)	
Recurrent urinary tract infection	10 (83)
Failed anti-reflux operation	2 (17)
Post-op hospital stays (d), median (range)	3 (2-5)
Follow-up (m), median (range)	22 (13-63)

*VUR* vesico ureteral reflux

The HD grades of the ureteral orifice in relation to VUR grade and timing are summarized in Table 2. H3-dilation were presented in 12 ureters (75%) in the present cases. The portion of H3 ureters were increased as VUR grade increasing as VUR II: 40%; VUR III: 88%; 100%: VUR IV. Where filling VUR were pointed, H3 ureters were more evident: 78%; H2: 11%; H1; 11%.

**Table 2:** Numbers of affected ureters for each grade of hydrodistension and vesicoureteral reflux

HD grade	VUR grade			
	II	III	IV	Total
	(n = 5)	(n = 8)	(n = 3)	(n = 16)
H1	0 [0]	1 [1]	0 [0]	1 [1]
H2	3 [1]	0 [0]	0 [0]	3 [1]
H3	2 [0]	7 [4]	3 [3]	12 [7]
Total	5 [1]	8 [5]	3 [3]	16 [9]

*HD* hydrodistension grade, *VUR* vesicoureteral reflux

Vesicoureteral reflux timing is presented in brackets as, n [filling vesicoureteral reflux]

### Surgical procedure

The injection methods used in relation to HD grade and VUR grade were summarized in Table 3. I-HIT combined with STING method were conducted in 10 ureters (63%), following G-HIT combined with STING in 4 ureters (25%). The median number of injection sites (range) were, 4(3–6) points, 4(3–5) points, 3 points and 2 points, respectively. The median volume (range) were, 2.5 (1.2–3.0) ml, 2.0 (2.0–3.0) ml, 1.5 ml and 0.6 ml. These showed no significant differences between these 2 methods (*P* = 0.203 and *P* = 0.102, respectively). Exceptionally, in 2cases of H3 dilated ureter, G-HIT combined with STING method were used owing to distal ureteral deformities. In 1 case of H2 dilated ureter, was added the Dx/HA by the STING.

**Table 3:** Numbers of ureters according to selected injection methods for each grade of hydrodistension and vesicoureteral reflux

Injection method	HD grade			VUR grade		
	H1	H2	H3	II	III	IV
	(n = 1)	(n = 3)	(n = 12)	(n = 5)	(n = 8)	(n = 3)
I-HIT + STING			10	2	6	2
G-HIT + STING		2	2	2	1	1
G-HIT		1		1		
STING	1				1	

*HD* hydrodistension grade, *VUR* vesicoureteral reflux*, I-HIT* inserting hydrodistension implantation technique, *G-HIT* guide-wired hydrodistension implantation technique, *STING* subureteral transurethral injection

Intraoperative cystography showed VUR, in 3 of 4 cases of bilateral VUR confirmed preoperatively and 4 ipsilateral ureters from 8 patients with single-sided VUR. There were no cases of intraoperative VUR that had not been diagnosed preoperatively. There was not apparent correlation between postoperative and intraoperative VUR grade (data not shown)

### Surgical results

**Fig. 3:**
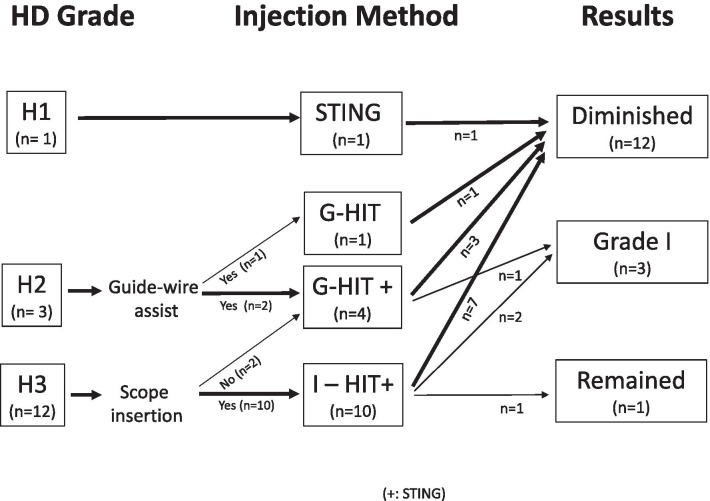
Flowchart of patient progress during the study. *HD* hydrodistension grade, *VUR* vesicoureteral reflux*, I-HIT* inserting hydrodistension implantation technique, *G-HIT* guide-wired hydrodistension implantation technique, *STING* subureteral transurethral injection

The flow chart of patients’ progress is presented in Fig. 3. Postoperatively, VUR was diminished to grad 0 in 12 ureters (75%) and decreased to grade I in 3 ureters (19%). In patients-based words, 9 (75%) cases succeeded and 11 (92%) cured after the-first operation. Only one case, who underwent the first operation via I-HIT combined with STING method, remained as VUR grade III perceived successful second injection therapy; no patients required a third injection.

The success rates of each injection methods in terms of HD and VUR grades were summarized in Table 4. I-HIT combined with STING method, only conducted in H3 dilated ureters, succeeded in 7 (70%) ureters. G-HIT combined with STING method was successed in 3 (75%), G-HIT: 1 (100) % and STING: 1 (100%) ureter. There was no statistical difference (*P* = 0.588).

**Table 4:** Numbers of succeeded ureters, n (%) according to each injection methods for each grade of hydrodistension and vesicoureteral reflux

Injection method	HD grade			VUR grade			*p *value ^#^
	H1	H2	H3	II	III	IV	
	(n = 1)	(n = 3)	(n = 12)	(n = 5)	(n = 8)	(n = 3)	
I-HIT + STING			7 (70)*	2 (100)	3 (50)*	2 (100)	0.588
G-HIT + STING		1 (50)**	2 (100)	1 (50)**	1 (100)	1 (100)	
G-HIT		1 (100)		1 (100)			
STING	1 (100)				1 (100)		

*HD* hydrodistension grade, *VUR* vesicoureteral reflux*, I-HIT* inserting hydrodistension implantation technique, *G-HIT* guide-wired hydrodistension implantation technique, *STING* subureteral transurethral injection

*One failed ‘dropped’ ureter, 1 downgraded ‘dropped’ ureter, and 1 downgraded ureter after collagen injection therapy

^**^One downgraded ‘kinked’ ureter

^#^Calculated by Fisher’s exact test

As for H3 dilated ureters, all of grade IV VUR were diminished apart from those in which the ‘filling reflux’ was present preoperatively, though 1 failed and 2 downgraded ureters with grade III VUR were exist. In 1 bilateral case with 1 failed and 1 downgraded ureter, initially diagnosed as grade III VUR with H3 dilation, treatment of both sides was carried out via this method; one side remained grade III and another was downgraded to grade I postoperatively. Intraoperative cystoscopy had showed bilateral deformity of the distal ureter (‘dropped ureters’) because of large myoma or flaccid bladder. In the second operation, these were treated successfully via the same method, but injecting into more lateral portion of the intramural ureter where a sufficient mound was formed due to the softness of the tissue. The other case remained grade I VUR ureter exhibited insufficient mound height due to tissue stiffness owing to a history of collagen injection therapy. Another 2 cases with H3 dilated ‘dropped ureter’, one with grade IV and one with grade III VUR, were exceptionally treated via G-HIT combined with STING in order to inject into lateral portion of the distal ureter, where softness of the tissue was suspected, and resulted successful outcomes.

As for H2 cases, 1 patient had a history of laparoscopic implantations by extravesical approach and had remained grade II VUR was treated successfully via G-HIT combined with STING method. Although the other remained grade I VUR case, lateral lifting (‘kinking’) of the H2 dilated ureter due to deviation of the uterus lead to insufficient mound height.

### Complications and comorbidity

Temporary postoperative mild HN (G1 or G2) occurred in three ureteral units (19%) at 1 month postoperatively. These HN disappeared until 3 months without any urinary-tract infection. Dull flank pain was reported by three patients for several days postoperatively, which was safely managed with painkillers like acetaminophen. Only one case experienced acute left-sided pyelonephritis requiring antibiotic infusion for 3 days before discharge. In this case, contralateral right-side ureter was treated first, although intraoperative cystography showed grade I VUR in left side. During follow-up duration; median 23 (range 13–63) months long, 9 patients were suffered no f-UTI. Although, 3 patients were experienced f-UTI 1–2 times, repeated VCUG showed no VUR recurrence.

In one of 12 cases (6%), contralateral VUR (i.e., occult VUR) newly recognized grade IV was detected after 1.5 years of follow-up without any f-UTI, while intraoperative cystoscopy had detected H2 dilation of ureteral orifices and lateral positioning.

## Discussion

The present study demonstrates that endoscopic injection therapy is an effective and safe treatment for VUR in post-pubertal female patients. Conducted via modified HIT techniques adopted for dilated ureteral orifice, we achieved successful results in 75% of patients and over 90% of patients were cured after the first operation. In the present study, although post-pubertal female patients had some risks to injection therapy i.e. ureteral deformity or history of anti-reflux surgery that cause tissue stiffness, our meticulous method to select adequate site in the dilated ureter overcame those risks.

A previous meta-analysis has shown that refinement of injection materials and methods has led to the present success rate over 70% in children [15]. Recently, by a single experienced pediatric urologist, HIT has been reported to have a high success rate in adult [16], although this was among patients with mainly low-grade VUR. The present study demonstrates high success rates even in grade IV filling VUR applying modified HIT techniques adopted for adult ureters.

Reported predictors for success include a volcano-like mound appearance [17], mound height (as assessed by ultrasound) [18] and injection volume [19]. Moreover, VUR grade [20] and timing of VUR are suspected predictors [21]. And also, HD grade could be another predictor, seeing the fact HD grade is reported to have high correlation with VUR grade [13], in accordance with our present results. In adult ureter, additional risks are exist seeing the present study, like that the deformity (‘dropped’ or ‘kinked’) and the pathological stiffness owing to historical change more evident in adult ureters.

To manage these requirements, the injection must be conducted carefully with the needle align with the ureter puncturing at the adequate location where good tissue-softness guaranteed, so as to ensure enough mound appearance and height. We used two kinds of methods, I-HIT or G-HIT, with technique-selection according to HD grade adopted for large-caliber adult ureter. As a result, we found high success rate even in grade IV VUR. The flow chart that we developed and is presented here may provide a guide for the selection of injection technique.

In the present study, we found ureteral deformities or a history of anti-reflux surgery were associated with reduced success of outcomes. Anatomical anomalies like complete duplicated ureter pose a challenge to injection [22]; therefore, ureterocele and Hutch diverticulum are considered contraindications for endoscopic injection therapy [23]. In these complicated ureters, care must be taken to inject with an adequate volume to create sufficient mound height. These risks may be ameliorated using meticulous methods like I-HIT or G-HIT.

In the present study, we have experienced one case of acute pyelonephritis requiring antibiotic infusion before discharge. In this case, single sided VUR confirmed by intraoperative cystography may lead to subsequent f-UTI. Maintaining bladder volume and considering the intraoperative VUR could result in better outcomes. In this meaning, intraoperative cystography has some benefit.

This study showed the prevalence of occult VUR to be considerable postoperatively (6%). Although intraoperative cystography is not recommended for detection of occult VUR owing to its low sensitivity [24], other methods to predict occult VUR are needed. Alternatively, protective injection according to HD grade could be recommended [25], so that VUR grade is reported to have high correlation with HD grade [21] as mentioned in the former section.

We recommend 6 months of follow-up including ultrasound examination after surgery, based on our finding that temporary postoperative HN until 3 months occurred in nearly 20% of patients. One of the risks of obstruction, the beak sign of ureterovesical junction [16], may have contributed to any case of persistent HN.

The present study had some limitations which should be acknowledged. First, this was a retrospective study with a small sample size. However, we included all consecutive patients, and all operations were performed by a single urologist at a single institution, thereby reducing bias relating to the cohort. Secondly, not all patients underwent repeated VCUG after successful treatment. Although late recurrence was reported in 20% of cases over the 2 years [26–28]. Three-month postoperative VCUG examinations are widely performed while late VCUG is not routinely performed due to the radiation exposure involved [29]. We performed regular urinalysis and ultrasound postoperatively and would recommend that repeated VCUG is performed in cases where at least one episode of f-UTI or repeated afebrile UTI are experienced. Long-term follow-up including ultrasound or dimercaptosuccinic acid examination of growth and blood pressure is important for patients with renal scarring [30]. Further randomized clinical trials with larger cohorts evaluating long-term clinical outcomes, prevention of f-UTI, and renal function are required to fully confirm the efficacy and safety of injection therapy in adult patients.

## Conclusions

In this study, we have showed that endoscopic injection therapy can achieve a high success rate with few complications even in post-pubertal female patients. While ureteral deformities or a history of anti-reflux surgery may increase the risks, these can be managed with appropriate methods that ensure sufficient mound appearance and height. Via modified HIT techniques according to HD grade of the ureteral orifice, endoscopic injection therapy is an effective and safe treatment for VUR in post-pubertal female patients.

## Data Availability

All datasets collected and analyzed in this study will be available by the corresponding author upon any reasonable request.

## Abbreviations

Dx/HA: Dextranomer/hyaluronic acid copolymer
f-UTI: Febrile urinary tract infection
HIT: Hydrodistension implantation technique
HD: Hydrodistension
STING: Subureteral transurethral injection
VUR: Vesicoureteral reflux
VCUG: Voiding cystourethrography
